# Effectiveness of a Mobile App (KhunLook) Versus the Maternal and Child Health Handbook on Thai Parents’ Health Literacy, Accuracy of Health Assessments, and Convenience of Use: Randomized Controlled Trial

**DOI:** 10.2196/43196

**Published:** 2023-05-09

**Authors:** Rosawan Areemit, Suchaorn Saengnipanthkul, Sumitr Sutra, Pagakrong Lumbiganon, Phenphitcha Pornprasitsakul, Pongsatorn Paopongsawan, Kunwadee Sripanidkulchai

**Affiliations:** 1 Department of Pediatrics Faculty of Medicine Khon Kaen University Khon Kaen Thailand; 2 Department of Computer Engineering Faculty of Engineering Chulalongkorn University Bangkok Thailand

**Keywords:** health literacy, mobile app, mHealth, KhunLook, Maternal and Child Health Handbook, parent, health assessment, child health supervision, Thailand

## Abstract

**Background:**

Children of parents who have higher health literacy (HL) benefit more from preventive child health care. Digital interventions have been used to improve parents’ HL with high satisfaction. KhunLook is a Thai mobile app conceived using strategies to improve HL. It was developed to assist parents in assessing and keeping track of their child’s health in complement to the standard Maternal and Child Health Handbook (MCHH).

**Objective:**

This trial focuses on the effectiveness of using the KhunLook app with the MCHH and standard care (intervention) compared with the conventional MCHH and standard care (control) on parents’ HL. Data on accuracy of parents’ assessment of their child’s health and growth as well as convenience of use of the tool (app or MCHH) in the well-child clinic were collected at 2 visits (immediate=visit 1, and intermediate=visit 2).

**Methods:**

Parents of children under 3 years of age who (1) had a smartphone or tablet and the MCHH and (2) could participate in 2 visits, 2-6 months apart at Srinagarind Hospital, Khon Kaen, Thailand, were enrolled in this 2-arm parallel randomized controlled trial between April 2020 and May 2021. Parents were randomized 1:1 to 2 groups. At visit 1, data on demographics and baseline HL (Thailand Health Literacy Scales) were collected. Parents in the app group used the KhunLook app and the control group used their child’s handbook to assess their child’s growth, development, nutrition and feeding, immunization status and rated the convenience of the tool they used. At visit 2, they repeated the assessments and completed the HL questionnaire.

**Results:**

A total of 358 parents completed the study (358/408, 87.7%). After the intervention, the number of parents with high total HL significantly increased from 94/182 (51.6%) to 109/182 (59.9%; 15/182; Δ 8.2%; *P*=.04), specifically in the health management (30/182; Δ 16.4%; *P*<.001) and child health management (18/182; Δ 9.9%; *P*=.01) domains in the app group, but not in the control group. Parents in the app group could correctly assess their child’s head circumference (172/182, 94.5% vs 124/176, 70.5%; *P*<.001) and development (173/182, 95.1% vs 139/176, 79.0%; *P*<.001) better than those in the control group at both visits. A higher proportion of parents in the app group rated their tool as very easy or easy to use (174-181/182, 95.6%-99.5% vs 141-166/176, 80.1%-94.3%; *P*<.001) on every item since the first visit.

**Conclusions:**

Our results suggest the potential of a smartphone app (KhunLook) to improve parents’ HL as well as to promote superior accuracy of parents’ assessment of their child’s head circumference and development, with a similar effect on weight, height, nutrition and feeding, and immunization as in traditional interventions. Using the KhunLook app is useful and more convenient for parents in promoting a healthy child preventive care during early childhood.

**Trial Registration:**

Thai Clinical Trials Registry TCTR20200312003; https://www.thaiclinicaltrials.org/show/TCTR20200312003

## Introduction

Development of the future population depends on the health of today’s children. Investment in preventive health such as child health supervision since early childhood results in better health outcomes [1,2]. Child health supervision focuses on engaging parents as partners throughout its trajectory of health care services such as assessment of growth, development, nutrition and feeding, and immunizations [2]. This makes parents and primary caregivers vital to maximizing child health [2,3].

Parents’ health literacy (HL) affects their ability to use health information to make health decisions for their children [1,4]. Studies have identified that children of parents who have higher HL benefit more from preventive child health care because they are more likely to understand critical aspects of pediatric anticipatory guidance and have better access to preventive care services [4-9]. In the United States and Germany, 1 out of 2-4 parents have low HL [4,9]. A study in Thailand found that 16.8% of Thai adults had high HL [10]. Encouragingly, HL is not a fixed attribute and can be improved by interventions [11-13].

Digital interventions have been used to improve parents’ HL with high satisfaction [13]. Mobile health (mHealth) technology interventions are highly potential because they can be tailored to users in various health care settings; designed with a universal approach to HL; and can retain simplicity, accessibility, portability, and scalability [13-15]. Mobile apps for maternal and child health are increasingly developed worldwide [16-20]. Although the outcomes from a meta-analysis showed mixed effects [21], there are increasing numbers of ongoing studies on mHealth interventions for maternal and child health [19,20].

While more parents turn to online resources including mobile apps for childcare, apps have been critiqued for credibility and quality [21-26]. Much of the criticisms arise from not listing a source for the information provided, issues with engagement, aesthetics, and incorporating commercial ads [21-26]. In addition, many apps are in English which may not be fully useful in countries that do not use English as a first language such as Thailand.

KhunLook is a Thai mobile app developed to assist parents in assessing and keeping track of their child’s health in complement to the Maternal and Child Health Handbook (MCHH) [27]. The MCHH is widely used and serves as the standard personal child health supervision physical record [28-30]. Despite having comprehensive information, the MCHH is infrequently read [27,31]. The KhunLook app was designed to interactively meet user’s needs and was developed by an interdisciplinary team of medical and dental specialists, software engineers, health care providers, and families. It was released in 2015 and follows the Royal College of Pediatricians of Thailand and Ministry of Public Health’s health supervision guidelines.

KhunLook was conceived with HL-informed strategies such as simplifying, chunking, organizing, and limiting information as well as including short written information and picture graphics [4,27]. A pilot study assessed the feasibility and accuracy of parents’ assessments of their child’s growth parameters and found that it was well accepted. A national scale survey also reported that KhunLook was significantly easier to use than the MCHH [27]. Assessment of implementing the KhunLook app in the well-child clinic and its effects of on parents’ HL and accuracy of their assessments on other functions (eg, child development, nutrition and feeding, and immunizations) have yet to be determined.

This trial focuses on the effectiveness of using the KhunLook app with the MCHH and standard care (intervention) compared with the conventional MCHH and standard care (control) on parents’ HL, immediate (visit 1) and intermediate (visit 2) accuracy of parents’ assessment of their child’s health in addition to growth, and convenience of use in the well-child clinic.

## Methods

### Study Design

This 2-arm parallel randomized controlled trial was conducted between April 2020 and May 2021 at the Well-Child Clinic, Pediatric Outpatient Department, Srinagarind Hospital, Faculty of Medicine, Khon Kaen University, North-East Thailand.

### Ethics Approval

The trial was registered in the Thai Clinical Trials Registry (TCTR20200312003) and approval for this study was obtained from the Research Ethics Committee, Khon Kaen University (HE561373)

### Recruitment

Although the term “parent” will be used throughout this article, this term includes guardians, additional family, or individuals that provide care to children who bring them to well-child visits. Parents were recruited during routine child health supervision visits. They received written information about the study and those who were interested in participating contacted the research team. Posters were used to promote the study; participation was voluntary and written consent from parents was obtained. Inclusion criteria were (1) being a parent of child who was younger than 3 years, (2) having a smartphone or a tablet with iOS or Android operating system, and (3) having the MCHH and can read and answer the questionnaires. Parents were required to participate in 2 visits, 2-6 months apart depending on the child’s next health supervision schedule. Parents who could not be present at both visits were excluded.

### Sample Size, Randomization, and Blinding

We estimated that 356 parents (178 in each group) were needed, considering our hypothesis that using the KhunLook app could decrease the proportion of parents with low to fair HL from 83.2% by 15% when compared with the MCHH with a power of 80% and a significance level of 5%. Considering possible loss to follow-up, we recruited just over 400 parents. We used a computer-based block-of-4 randomization list to allocate parents at a 1:1 ratio to either the control group or the intervention group. Allocation concealment was ensured using opaque envelopes (Piangjit Tharnprisan), which was opened by the research assistant (Marisa Klangkhumpa) after completion of informed consent whereby the parent was informed of their allocation group. The parents were not blinded to the allocation due to the nature of the intervention.

### Data Collection

**Figure 1:**
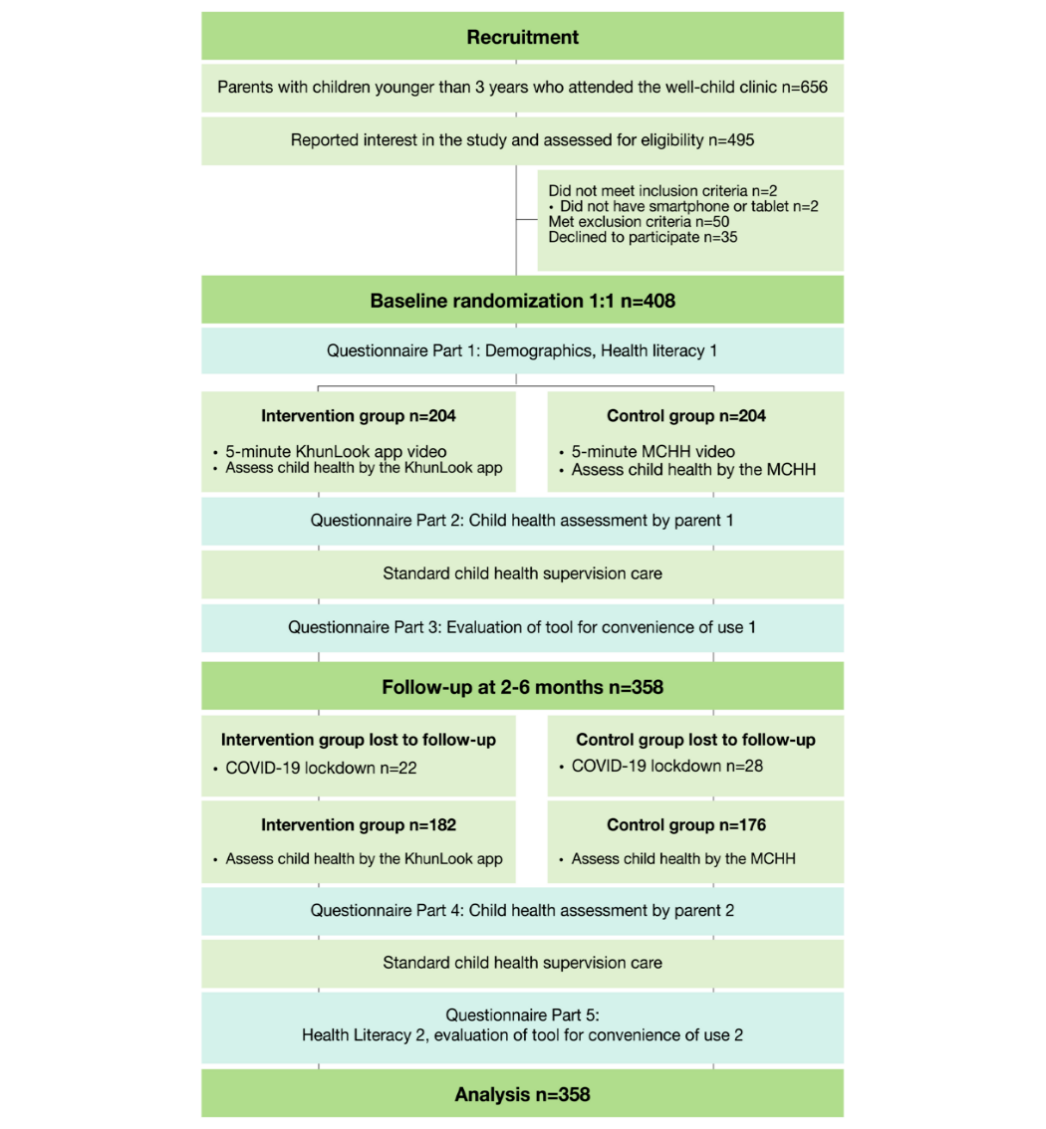
CONSORT (Consolidated Standards of Reporting Trials) flowchart of the trial. HL: health literacy; MCHH: Maternal and Child Health Handbook.

The sequence of data collection is shown in Figure 1 (also see Multimedia Appendix 1). At visit 1, parents completed a self-administered questionnaire on demographics and baseline HL.

Both groups watched a 5-minute video on health assessment guidance using the KhunLook app or the MCHH. The videos were in Thai language, had matching content, and a demonstration of how to use either the KhunLook app (intervention group) or the MCHH (control group). Children’s growth parameters were measured by the health care staff and were provided to parents. Parents in the app group used the KhunLook app version 4.1 for iOS or version 4.1 for Android (equivalent in functionality); there were no changes in app versions during the trial. Parents in the MCHH group used their child’s handbook to assess their child’s growth, development, nutrition and feeding, and immunization status before meeting the physician. Physicians provided standard child health supervision, which included assessment of growth, development, and immunization status. Physicians were blinded to the parents’ group allocation, parents’ assessment of child’s health, and did not use the app. After visiting the physician, parents rated the convenience of the tool they used.

At visit 2, children’s growth parameters were measured and provided to parents. Parents assessed their child’s growth, development, nutrition and feeding, and immunization status before meeting the physician using the same tool as in the first visit, without guidance. After visiting the physician, parents completed the HL questionnaire and rated the convenience of the tool they used.

### Instruments

We used a questionnaire to collect the baseline characteristics. The primary outcome was the decrease in the proportion of parents with low to fair HL. The secondary outcomes were changes in HL scores, accuracy of health assessment by parents, and convenience of use.

We used the Thailand Health Literacy Scales, a generic, 47-item, 5-point Likert scale questionnaire to evaluate parents’ HL [10]. Parents ranked their agreement to each item from very unlikely (score=1) to very likely (score=5). Total HL is a summary score calculated from 5 domains. The scale was developed for assessment of HL in the Thai general healthy population and has been used with 7- to 75-year-olds with a Cronbach α of .95-.97 [10]. To make the scale more specific to child health, we developed an additional domain (child health management) by rephrasing items from the health management domain. The word “health” in each item was replaced by “child health.” This domain has been used with Thai parents with good internal consistency (Cronbach α=.94) [32].

Outcomes of child health assessment included weight, height/length, head circumference (below normal, normal, and above normal), development (delayed or normal), nutrition and feeding (inappropriate or appropriate), and immunization status (delayed or up to date). Growth standards were based on the Thai national growth reference [33], assessed in relation to sex and chronological age for term infants, and in relation to corrected age for preterm infants. Assessment of development, nutrition and feeding, and immunization status was based on the Royal College of Pediatricians of Thailand and Ministry of Public Health’s health supervision guidelines [34-36].

Parents rated the convenience of using the intervention (KhunLook app) or the control (MCHH) on 12 domains on a 4-rank rating scale (very easy, easy, difficult, and very difficult).

### Statistical Analysis

Demographics were analyzed using descriptive statistics. The 2-sided unpaired *t* test and the chi-square test were used, when appropriate.

HL scores were calculated, and parents’ HL category was considered “high” when scores equaled 80% or more, “fair” when scores equaled 60% to less than 80%, and “low” when scores were less than 60% for the total scale and for all domains [10]. The McNemar test was used to determine differences in proportions of parents with high HL versus fair to low HL, and the paired *t* test was used to determine the differences between HL scores at baseline and visit 2 within groups. The chi-square test was used to compare the proportions of parents with high HL versus fair to low HL, and the unpaired *t* test was used to determine the differences between HL scores between the control and intervention groups. Cohen *d* was used to determine the effect sizes of the mean score differences.

The physician’s assessment of the child’s health was used as the standard. Parents’ health assessments that matched the physician’s assessment were considered accurate. The chi-square test was used to determine the differences between the proportions of parent-physician congruence of child health assessments between the app group and the MCHH group. The McNemar test was used to determine the differences in proportions of parent-physician congruence of child health assessments at baseline and visit 2 within groups.

For convenience of use, ratings were first grouped into “very easy to easy” and “difficult to very difficult,” and then proportions were calculated. The Pearson chi-square or Fisher exact test was used to determine the differences between the proportions of convenience of use between both groups.

We considered possible differences between subgroups and conducted a post hoc subgroup analysis in parents who were male, single, and had more than 1 child. The rationale is that males may be more willing to adopt mHealth interventions, single parents who may have less support could have more incentive to use apps, and parents could have been previously exposed to the app and MCHH if the child included was not the first child. For the subgroup analysis, we used the Bonferroni correction to adjust the α for significance, given 4 subgroups regarding parental status (parents of the 1st child, parents have more than 1 child, single parents, and parents who are men), with 2 groups (intervention vs control). An adjusted α is considered statistically significant when the *P* value is <.00625. The STATA Statistical Software (release 14; StataCorp LP) was used for the statistical calculations [37].

## Results

### Overview

During the time of the study there were 656 parent-child pairs who met the age criteria. Among these, 495 contacted the team; however, 50 were moving and thus could not come for the second visit, 2 did not have a mobile device, and 35 parents declined participation (Figure 1).

Of the 408 parents who participated, 204 were allocated to each group. Because of lockdowns during the COVID-19 pandemic, 50 participants could not come for the second visit. A total of 358 parents completed the study (Figure 1). The baseline characteristics of parents and children are shown in Table 1. Most parents were female (316/358, 88.3%), married (308/358, 86.0%), and 63.4% (227/358) of the children were the first child. There were no statistically significant differences between the parents’ characteristics in both groups except for their mean age (*P*=.007), which was 1.9 years less in the app group (Table 1).

**Table 1:** Demographics of parents.

Demographics					KhunLook app group (n=182)			MCHH^a^ group (n=176)			*P* value			
**Parent factors**														
	**Sex,** **n (%)**									.15				
		Female	165 (90.7)			151 (85.8)								
		Male	17 (9.3)			25 (14.2)								
	Age (years), mean (SD); range			31.2 (6.3); 15-58			33.1 (7.4); 17-63			.007				
	**Status, n (%)**									.68				
		Single	22 (12.1)			22 (12.5)								
		Married	158 (86.8)			150 (85.2)								
		Other	2 (1.1)			4 (2.3)								
	**Parents of first child, n (%)**									.31				
		Yes	120 (65.9)			107 (60.8)								
		No	62 (34.1)			69 (39.2)								
	**Education, n (%)**									.20				
		Less than bachelor’s degree	72 (39.6)			60 (34.1)								
		Bachelor’s degree	96 (52.7)			93 (52.8)								
		Master’s or doctoral degree	14 (7.7)			23 (13.1)								
	Household income <15,000 Thai Baht^b^/month, n (%)			41 (22.5)			52 (29.6)			.13				
	**Type of phone, n (%)**									.41				
		iPhone	59 (32.4)			50 (28.4)								
		Android	123 (67.6)			126 (71.6)								
**Child factors**														
	**Sex,** **n (%)**									.35				
		Female	98 (53.8)			86 (48.9)								
		Male	84 (46.2)			90 (51.1)								
	Age (years), mean (SD); range			0.87 (0.59); 0.17-2.91			0.87 (0.62); 0.08-2.85			.99				
	**Child order**									.20				
		1	120 (65.9)			107 (60.8)								
		2	50 (27.5)			62 (35.2)								
		3	11 (6.0)			5 (2.8)								
		4	1 (0.5)			2 (1.1)								
	Were born at gestational age ≥ 37 weeks, n (%)			158 (86.8)			153 (86.9)			.97				
	Have an underlying disease, n (%)			13 (7.1)			13 (7.4)			.93				
	Child’s MCHH version (year), range			2012-2020			2012-2020							
	Time interval between visits 1 and 2 (months), mean (SD)			2.69 (1.26)			2.72 (1.68)			.80				

^a^MCHH: Maternal and Child Health Handbook.

^b^1 Thai Baht=US $0.029.

### Parents’ HL

**Figure 2:**
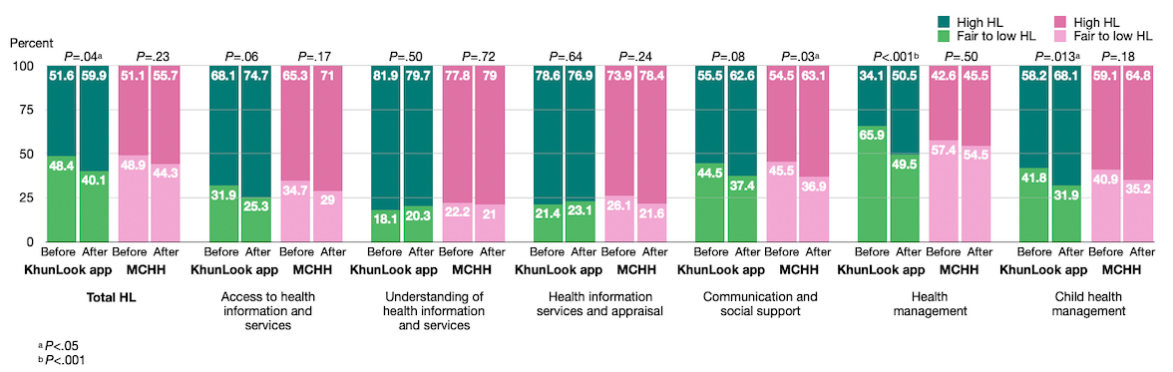
Proportions of parent’s health literacy by category before and after the intervention. HL: health literacy; MCHH: Maternal and Child Health Handbook.

The proportion of parents’ HL category (high vs fair to low) is shown in Table 2 and Figure 2. Baseline proportions of the HL category were not significantly different between groups at both visits (*P*=.93 for total HL at visit 1 and *P*=.42 at visit 2). After the intervention, the proportion of parents with high HL increased, and the proportion of parents with fair to low HL decreased in the app group for the total HL (15/182; Δ 8.2%; *P*=.04), health management (30/182; Δ 16.4%; *P*<.001), and child health management (18/182; Δ 9.9%; *P*=.01) domains with statistical significance. The proportion of parents with high HL increased, and the proportion of parents with fair to low HL decreased with statistical significance in the MCHH group in the communication and social support domain (15/176; Δ 8.5%; *P*=.03).

Parents’ mean HL scores are shown in Table 3. The baseline and final mean scores did not differ between groups. After the intervention, mean scores significantly increased in both groups, for total HL (app group, *P*=.003; MCHH group, *P*=.01) and 4 domains, namely, access to health information and services (app group, *P*=.02; MCHH group, *P*=.04); health communication and social support (app group, *P*=.008; MCHH group, *P*=.005); health management (app group, *P*<.001; MCHH group, *P*=.02); and child health management (app group, *P*=.002; MCHH group, *P*=.02). The effect size for domains of HL that significantly increased was small (Cohen *d*=0.14-0.30). Most effect sizes were slightly larger in the app group when compared with the MCHH group. The internal consistency of the Thailand Health Literacy Scales and the rephrased “child health management” domain in this study was excellent (Cronbach α=.97-.98 for total scale and .93-.96 for the child health management domain).

**Table 2:** Proportions of parents’ health literacy by category before and after the intervention.

Health literacy domain and category			KhunLook app group (n=182)							MCHH^a^ group (n=176)				
			Before, n (%)		After, n (%)		*P* value		Before, n (%)			After, n (%)		*P* value
**Total health literacy**							.04							.23
	High	94 (51.6)		109 (59.9)				90 (51.1)			98 (55.7)			
	Fair to low	88 (48.4)		73 (40.1)				86 (48.9)			78 (44.3)			
**Access to health information and services**							.06							.17
	High	124 (68.1)		136 (74.7)				115 (65.3)			125 (71.0)			
	Fair to low	58 (31.9)		46 (25.3)				61 (34.7)			51 (29.0)			
**Understanding of health information and services**							.51							.72
	High	149 (81.9)		145 (79.7)				137 (77.8)			139 (79.0)			
	Fair to low	33 (18.1)		37 (20.3)				39 (22.2)			37 (21.0)			
**Health information services and appraisal**							.64							.24
	High	143 (78.6)		140 (76.9)				130 (73.9)			138 (78.4)			
	Fair to low	39 (21.4)		42 (23.1)				46 (26.1)			38 (21.6)			
**Communication and social support**							.08							.03
	High	101 (55.5)		114 (62.6)				96 (54.5)			111 (63.1)			
	Fair to low	81 (45.1)		68 (37.4)				80 (45.5)			65 (36.9)			
**Health management**							<.001							.5
	High	62 (34.1)		92 (50.5)				75 (42.6)			80 (45.5)			
	Fair to low	120 (65.9)		90 (49.5)				101 (57.4)			96 (54.5)			
**Child health management**							.01							.18
	High	106 (58.2)		124 (68.1)				104 (59.1)			114 (64.8)			
	Fair to low	76 (41.8)		58 (31.9)				72 (40.9)			62 (35.2)			

^a^MCHH: Maternal and Child Health Handbook.

**Table 3:** Parents’ health literacy scores before and after the intervention.

Health literacy domain	KhunLook app group (n=182)					MCHH^a^ group (n=176)			
	Before, mean (SD)	After, mean (SD)	*P* value	*d*	Before, mean (SD)		After, mean (SD)	*P* value	*d*
Total health literacy	189.5 (22.4)	193.7 (24.1)	.003	0.18	188.6 (24.3)		192.3 (23.7)	.01	0.15
Access to health information and services	33.3 (3.8)	33.9 (4.0)	.02	0.17	32.9 (4.5)		33.4 (4.0)	.04	0.14
Understanding of health information and services	21.3 (2.5)	21.4 (2.7)	.58	N/A^b^	21.1 (2.8)		21.1 (2.8)	.98	N/A
Health information services and appraisal	25.5 (3.1)	25.2 (3.3)	.18	N/A	25.2 (3.0)		25.3 (3.1)	.94	N/A
Communication and social support	68.3 (10.3)	70.0 (10.1)	.008	0.18	67.8 (10.9)		69.7 (10.3)	.005	0.19
Health management	41.2 (6.6)	43.2 (6.9)	<.001	0.30	41.7 (6.9)		42.7 (6.6)	.02	0.16
Child health management	44.5 (5.8)	45.9 (6.2)	.002	0.23	44.4 (6.0)		45.4 (6.2)	.02	0.17

^a^MCHH: Maternal and Child Health Handbook.

^b^N/A: not applicable.

### Accuracy of Parents’ Assessment of Child Health

The congruence of child health assessments by parents and physicians is shown in Tables 4 and 5. Parents in the app group could correctly assess their child’s head circumference and development better than those in the MCHH group with statistical significance at both visits (Table 4). The proportion of correctness of other health domain assessments was similar between both groups. The proportion of correct assessments in the app group was already high (156-179/182, 85.7%-98.4%) at the first visit and did not significantly change at the second visit (Table 5). The proportion of correct assessments in the MCHH group was generally lower (124-167/176, 70.5%-94.9%) at the first visit, and significantly improved for 1 component (head circumference assessment, *P*=.001) at the second visit.

**Table 4:** Accuracy of parents’ assessment of their child’s health compared by group.

Visit and assessment parameters			KhunLook app group (n=182)							MCHH^a^ group (n=176)							*P* value
			Parent, normal assessment, n (%)		Physician, normal assessment, n (%)		Accuracy of parents’ assessment, n (%)		Parent, normal assessment, n (%)			Physician, normal assessment, n (%)		Accuracy of parents’ assessment, n (%)			
**Visit 1**																	
	Weight	162 (89.0)		162 (89.0)		159 (87.4)		151 (85.8)			143 (81.3)		147 (83.5)		.30		
	Length/height	156 (85.7)		166 (91.2)		156 (85.7)		152 (86.4)			161 (91.5)		154 (87.5)		.62		
	Head circumference	175 (96.2)		173 (95.1)		172 (94.5)		122 (69.3)			166 (94.3)		124 (70.5)		<.001		
	Development	180 (98.9)		175 (96.2)		173 (95.1)		142 (80.7)			168 (95.5)		139 (79.0)		<.001		
	Immunization	180 (98.9)		175 (96.2)		179 (98.4)		168 (95.5)			173 (98.3)		167 (94.9)		.07		
	Feeding	180 (98.9)		168 (92.3)		167 (91.8)		172 (97.7)			158 (89.8)		156 (88.6)		.32		
**Visit 2**																	
	Weight	167 (91.8)		157 (86.3)		160 (87.9)		157 (89.2)			152 (86.4)		155 (88.1)		.96		
	Length/height	162 (89.0)		167 (91.8)		159 (87.4)		154 (81.5)			165 (93.8)		155 (88.1)		.84		
	Head circumference	174 (95.6)		176 (96.7)		170 (93.4)		151 (85.8)			166 (94.3)		148 (84.1)		.005		
	Development	178 (97.8)		171 (94.0)		169 (92.9)		162 (92.0)			159 (90.3)		148 (84.1)		.009		
	Immunization	175 (96.2)		182 (100)		176 (96.7)		169 (96.0)			175 (99.4)		168 (95.5)		.74		
	Feeding	171 (94.0)		164 (90.1)		158 (86.8)		163 (92.6)			156 (88.6)		148 (84.1)		.47		

^a^MCHH: Maternal and Child Health Handbook.

**Table 5:** Accuracy of parents’ assessment of their child’s health compared by visit.

Group	Assessment parameter	Visit 1				Visit 2			*P* value	
		Parent, normal assessment, n (%)	Physician, normal assessment, n (%)	Accuracy of parents’ assessment, n (%)	Parent, normal assessment, n (%)		Physician, normal assessment, n (%)	Accuracy of parents’ assessment, n (%)		
**App group (n=182)**										
	Weight	162 (89.0)	162 (89.0)	159 (87.4)	167 (91.8)		157 (86.3)	160 (87.9)	.86	
	Length/height	156 (85.7)	166 (91.2)	156 (85.7)	162 (89.0)		167 (91.8)	159 (87.4)	.62	
	Head circumference	175 (96.2)	173 (95.1)	172 (94.5)	174 (95.6)		176 (96.7)	170 (93.4)	.56	
	Development	180 (98.9)	175 (96.2)	173 (95.1)	178 (97.8)		171 (94.0)	169 (92.9)	.39	
	Immunization	180 (98.9)	175 (96.2)	179 (98.4)	175 (96.2)		182 (100)	176 (96.7)	.16	
	Feeding	180 (98.9)	168 (92.3)	167 (91.8)	171 (94.0)		164 (90.1)	158 (86.8)	.11	
**MCHH^a^ group (n=176)**										
	Weight	151 (85.8)	143 (81.3)	147 (83.5)	157 (89.2)		152 (86.4)	155 (88.1)	.17	
	Length/height	152 (86.4)	161 (91.5)	154 (87.5)	154 (81.5)		165 (93.8)	155 (88.1)	.85	
	Head circumference	122 (69.3)	166 (94.3)	124 (70.5)	151 (85.8)		166 (94.3)	148 (84.1)	.001	
	Development	142 (80.7)	168 (95.5)	139 (79.0)	162 (92.0)		159 (90.3)	148 (84.1)	.20	
	Immunization	168 (95.5)	173 (98.3)	167 (94.9)	169 (96.0)		175 (99.4)	168 (95.5)	.80	
	Feeding	172 (97.7)	158 (89.8)	156 (88.6)	163 (92.6)		156 (88.6)	148 (84.1)	.17	

^a^MCHH: Maternal and Child Health Handbook.

### Convenience of Use

Parents’ ratings of the tool they used to assess their child’s health (KhunLook app or MCHH) are shown in Tables 6 and 7. When compared between groups, a higher proportion of parents in the app group rated their tool as very easy or easy to use with statistical significance on every item at the first visit (*P*=<.001 to .009). Results between groups remained significant at the second visit, except *understandability of content* for which the difference was not statistically significant (Table 6). Convenience of use between visits on each tool was not significantly different, except the proportion of parents in the MCHH group who found that understandability of content increased at the second visit with statistical significance (Table 7).

**Table 6:** Convenience of use of tool compared by group.

Domain	Visit 1			Visit 2		
	KhunLook app group (n=182): rated very easy or easy, n (%)	MCHH^a^ group (n=176): rated very easy or easy, n (%)	*P* value	KhunLook app group (n=182): rated very easy or easy, n (%)	MCHH group (n=176): rated very easy or easy, n (%)	*P* value
Weight assessment	181 (99.5)	159 (90.3)	<.001	181 (99.5)	175 (99.4)	.001
Length/height assessment	179 (98.4)	155 (88.1)	<.001	178 (97.8)	158 (89.8)	.001
Head circumference assessment	179 (98.4)	145 (82.4)	<.001	177 (97.3)	150 (85.2)	<.001
Development assessment	178 (97.8)	141 (80.1)	<.001	179 (98.4)	158 (89.8)	.001
Immunization assessment	180 (98.9)	146 (83.0)	<.001	172 (94.5)	154 (87.5)	.004
Feeding assessment	178 (97.8)	147 (83.5)	<.001	169 (92.9)	155 (88.1)	.04
Data input	181 (99.5)	166 (94.3)	.009	179 (98.4)	161 (91.5)	.001
Access to desired part	174 (95.6)	155 (88.1)	<.001	173 (95.1)	157 (89.2)	.03
Understandability of content	175 (96.2)	147 (83.5)	<.001	176 (96.7)	165 (93.8)	.28
Applicability of content	177 (97.3)	155 (88.1)	<.001	176 (96.7)	160 (90.9)	.01
Usefulness	178 (97.8)	158 (89.8)	<.001	177 (97.3)	161 (91.5)	.01
Overall convenience	176 (96.7)	156 (88.6)	<.001	177 (97.3)	155 (88.1)	<.001

^a^MCHH: Maternal and Child Health Handbook.

**Table 7:** Convenience of use of tool compared by visit.

Domain	KhunLook app group (n=182)				MCHH^a^ group (n=176)		
	Visit 1: rated very easy or easy, n (%)	Visit 2: rated very easy or easy, n (%)	*P* value	Visit 1: rated very easy or easy, n (%)		Visit 2: rated very easy or easy, n (%)	*P* value
Weight assessment	181 (99.5)	181 (99.5)	.56	159 (90.3)		175 (99.4)	>.99
Length/height assessment	179 (98.4)	178 (97.8)	>.99	155 (88.1)		158 (89.8)	.71
Head circumference assessment	179 (98.4)	177 (97.3)	.71	145 (82.4)		150 (85.2)	.30
Development assessment	178 (97.8)	179 (98.4)	.41	141 (80.1)		158 (89.8)	.12
Immunization assessment	180 (98.9)	172 (94.5)	.16	146 (83.0)		154 (87.5)	.19
Feeding assessment	178 (97.8)	169 (92.9)	.16	147 (83.5)		155 (88.1)	.18
Data input	181 (99.5)	179 (98.4)	>.99	166 (94.3)		161 (91.5)	.18
Access to desired part	174 (95.6)	173 (95.1)	.16	155 (88.1)		157 (89.2)	.55
Understandability of content	175 (96.2)	176 (96.7)	.32	147 (83.5)		165 (93.8)	.001
Applicability of content	177 (97.3)	176 (96.7)	.66	155 (88.1)		160 (90.9)	.22
Usefulness	178 (97.8)	177 (97.3)	.56	158 (89.8)		161 (91.5)	.25
Overall convenience	176 (96.7)	177 (97.3)	.56	156 (88.6)		155 (88.1)	.83

^a^MCHH: Maternal and Child Health Handbook.

### Subgroup Analysis

There were 227/358 (63.4%) parents of the first child, 131/358 (36.6%) parents who had more than 1 child, 44/358 (12.3%) single parents, and 42/358 (11.7%) men. Details of the primary outcome by subgroup are presented in Multimedia Appendix 2.

For parents of the first child, after the intervention, the proportion of parents with high HL increased, and the proportion of parents with fair to low HL decreased in the app group in most domains. Specifically, the increase in the health management domain was statistically significant (*P*<.001). The changes in HL categories in the MCHH group were not enough to be statistically significant (*P*=.12 to >.99). The mean HL scores increased in both groups; in particular, the scores for the health management and child health management domains were statistically significant in the app group (*P*<.001 and .005, respectively). By contrast, in the MCHH group, the score for the increase in the communication and social support domain was increased (*P*=.005). Parents in the app group could correctly assess their child’s head circumference (first visit 112/120, 93.3% vs 71/107, 66.4%; *P*<.001; second visit 112/120, 93.3% vs 89/107, 83.2%; *P*=.02) and development (first visit 115/120, 95.8% vs 86/107, 80.4%; *P*<.001, second visit 112/120, 93.3% vs 86/107, 80.4%; *P*=.01) better than those in the MCHH group with statistical significance at the first visit. The proportions of correct assessments in the app group were already high at the first visit (app group 100-117/120, 83.3%-97.5%; MCHH group 71-104/107, 66.4%-97.2%) and did not significantly change at the second visit in the app group (*P*=.20 to >.99). Parents in the MCHH group could correctly assess their child’s head circumference at the second visit more than the first visit with statistical significance (MCHH correct head circumference assessment at the first visit was 73/107, 68.2%, whereas at the second visit it was 89/107, 83.2%; *P*=.003). When compared between groups, a higher proportion of parents in the app group rated their tool as very easy or easy to use on all items with statistical significance at the first visit (*P*<.002), except data input (*P*=.008). For the second visit, when compared between groups, a higher proportion of parents in the app group rated their tool as very easy or easy to use on all items; specifically, overall convenience remained statistically significant (*P*=.003).

Among parents who had more than 1 child, after the intervention, the proportion of those with high HL increased, whereas the proportion of those with fair to low HL decreased in the app group for the following domains: access to health information and services (*P*=.92), communication and social support (*P*=.83), health management (*P*=.32), and child health management (*P*=.11); however, the differences were not enough to be statistically significant. The proportion of parents with high HL increased, and the proportion of parents with fair to low HL decreased in the MCHH group in the following domains: access to health information and services, with statistical significance (12/69; Δ 17.4%; *P*=.003). The mean total HL scores increased in both groups, but the differences were not enough to be statistically significant (app group, *P*=.22; MCHH group, *P*=.56). Parents in the app group could correctly assess their child’s head circumference (60/62, 97% vs 53/69, 77%; *P*=.001), development (58/62, 94% vs 53/69, 77%; *P*=.008), and immunizations (62/62, 100% vs 63/69, 91%; *P*=.002) better than those in the MCHH group at the first visit. At the second visit, parents in the app group could correctly assess their child’s development better than those in the MCHH group (57/62, 92% vs 55/69, 80%; *P*=.05). The proportions of correct assessments in the app group were already high at the first visit (app group 56-62/62, 90%-100%; MCHH group 53-63/69, 77%-91%) and did not significantly change at the second visit (app group, *P*=.31 to >.99). Parents in the MCHH group could correctly assess their child’s weight at the second visit more than the first visit (first visit 56/69, 81%; second visit 64/69, 93%; *P*=.03). When compared between groups, a higher proportion of parents in the app group rated their tool as very easy or easy to use on all items at the first visit. Specifically, immunization assessment (*P*=.001), feeding assessment (*P*=.005), and understandability of content (*P*=.003) were statistically significant. For the second visit, when compared between groups, a higher proportion of parents in the app group rated their tool as very easy or easy to use on all items but the differences were not enough to be statistically significant (*P*=.007 to .4).

For single parents, after the intervention, the proportion of parents with high HL increased, and the proportion of parents with fair to low HL decreased in the app group for the total HL (*P*=.32), access to health information and services (*P*=.66), health management (*P*=.01), and child health management domains (*P*=.26), but the differences were not enough to be statistically significant. The proportion of parents with high HL increased, and the proportion of parents with fair to low HL decreased in the MCHH group in the communication and social support domain (*P*=.03), but the differences were not enough to be statistically significant. The mean total HL scores increased in both groups, but the differences were not enough to be statistically significant (app group, *P*=.65; MCHH group, *P*=.24). Single parents in the app group could correctly assess their child’s head circumference better than those in the MCHH at the first visit (22/22, 100% vs 17/22, 77%; *P*=.005). The proportions of correct assessments in the app group were already high at the first visit (app group 18-22/22, 82%-100%; MCHH group 17-21/22, 77%-96%) and did not significantly change at the second visit (app group 19-22/22, 86%-100%; MCHH group 18-22/22, 82%-100%; *P*=.03 to >.99). When compared between groups, a higher proportion of parents in the app group rated their tool as very easy or easy to use on every item at the first visit, but these differences were not enough to be statistically significant (*P*=.11 to >.99).

For male parents, the proportions of HL category were not significantly different between groups and at both visits (high total HL at baseline 25/42, 60%; after the intervention 22/45, 49%) and in all domains (*P*=.08 to >.99). The mean total HL scores increased in the app group (baseline 187.7; Δ 5.8; *P*=.14) more than those in the MCHH group (baseline 195.1; Δ 3.8; *P*=.35), but the differences were not enough to be statistically significant. Male parents in the app group could correctly assess their child’s head circumference better than those in the MCHH group at the first visit (16/17, 94% vs 16/25, 64%; *P*=.03). The proportions of correct assessments in the app group were already high at the first visit (app group 13-17/17, 77%-100%; MCHH group 16-24/25, 64%-96%) and did not significantly change at the second visit (app group 15-17/17, 88%-100%; MCHH group 19-23/24, 79%-96%; *P*=.06 to .74). When compared between groups, a higher proportion of parents in the app group rated their tool as very easy or easy to use on almost every item at the first visit, except access to desired part and understandability of content (app group 14/17, 82%; MCHH group 22/25, 88%), but these differences were not enough to be statistically significant (*P*=.21 to >.99).

## Discussion

### Principal Findings

This is the first randomized controlled trial to assess parents’ HL after using an app for child health assessments as part of routine child health supervision. We found that after using the KhunLook app, the proportion of parents who had high total HL significantly increased, whereas the proportion of parents with fair to poor HL decreased (15/182; Δ 8.2%). When looking at domains, the increase was significantly pronounced in the health management (30/182; Δ 16.5%) and child health management (18/182; Δ 9.9%) domains. Our findings are encouraging because the KhunLook app was designed to assist parents by providing high-quality information and making health assessments easier with the intention that parents would be able to detect abnormal growth and development in their children early on. Further, better HL in health management and child health management domains has implications for having good and enough health information to be able to manage health and prevent sickness, finding time to plan activities and choosing a better environment for better health, and participating in health activities with health care providers for themselves and their children [10].

We did not find similar changes in HL category shifts in parents who used the MCHH. By contrast, we found that using the MCHH significantly increased the proportion of parents with high HL in the communication and social support domain (15/176; Δ 8.5%; *P*=.03). This domain is related to having enough health support from their family, friends, and health care provider and being able to communicate for support [10]. This shift was also observed in the intervention group but with less magnitude (13/182; Δ 7.1%; *P*=.08). Perhaps the increase could be due to increased interaction with the health care team, because in routine care parents were not required to assess their child’s health with objective tools (KhunLook app or MCHH) before the visit.

We analyzed mean scores to allow for understanding of more subtle changes in HL, and found that parents’ scores significantly improved after the intervention in both groups for total HL and 4 subscales.

Our secondary outcomes show that parents in the intervention group could correctly assess their child’s head circumference and development better than those in the control group both immediately (visit 1) and intermediately (visit 2). More parents in the intervention group rated the app easier to use than those in the control group instantly at the first visit, with statistical significance in all domains. Upon follow-up, there were no significant changes in the proportion of parents who rated the app to be easy to use, suggesting sustained intermediate effects. Parents in the control group rated the content in the MCHH to become increasingly more understandable with statistical significance at visit 2. We propose that this may be because our study required parents to use the MCHH, and thus, they had a chance to engage with the MCHH more, while this was not required in routine care. In addition, participants in the intervention group were slightly younger than those in the control group and may have preferred a novel intervention over the conventional standard.

Looking into subgroups, we had 42 males in our study, 44 single parents, and 131 parents who had more than 1 child. The number of participants in each subgroup may not be enough to yield statistical power to make conclusions. However, the primary and secondary outcomes showed comparable trends to those from analysis of all parents. Our results tended to be more pronounced in parents of the first child. Parents who had more than 1 child could be previously exposed to the app, the MCHH (which may have different formats between versions), and child supervision from health care providers. This could lead to a possibility of a less accentuated effect of the outcomes. We found that parents who had more than 1 child and who were allocated to the MCHH group had a significant increase in the proportion of those with high HL in the “access to health information and services” domain. This could be because the MCHH, which is larger than a smartphone, could have created a perception of more information for this subgroup.

### Strengths and Limitations

This trial’s strengths are the randomized control design, high compliance (completion rate 358/408, 87.7%), implementation in a real-world clinical context, and the inclusion of parents from a wide range of educational levels and age. Our intervention was developed based on theoretical consideration of user-centered design with a universal approach to HL and with credible information [27]; additionally, the intervention is scalable and does not require intensive human interaction. The study also has limitations to be acknowledged, including the fact that it was carried out at a single institution and that the sample contained a larger proportion of parents with high HL than the general Thai adult population (184/358, 51.4% vs 168/1001, 16.8% [10]). This may influence generalizability to parents with lower HL status. The KhunLook app is already publicly available so parents in the control group could possibly also use the app. Nevertheless, we believe that our outcomes remain valuable due to the authenticity of the clinical context. Furthermore, an inherent limitation is the risk of recall bias and social desirability because a portion of our methods relied on self-report. Future research should therefore include parents from more subgroups and institutions, preferably from different regions of Thailand with a wider range of socioeconomic backgrounds.

### Comparison With Prior Work

Mobile apps are being developed to assist parents in monitoring their children’s health and have been evaluated for various child health outcomes [17-20,38-44], but parents’ HL has yet to be assessed. Our study shows that parents’ HL can be improved by using the KhunLook app which surpassed the standard MCHH in total HL and self- and child health management scales. Two studies have shown that apps can help improve parents’ understanding of growth charts [38] and in early detection of abnormal child growth [39]. A study involving 37 mothers found that an mHealth intervention had favorable, but without statistically significant, effects on child language development [40]. Beyond improving parents’ HL, our study indicates that the accuracy of parents’ evaluations of their child’s growth is increased when using the KhunLook app, which is significantly more accurate for head circumference and development in comparison to standard care, with immediate (visit 1) and intermediate (visit 2) effects. Previous studies indicated that parents found mHealth solutions to be satisfactory, highly convenient, and feasible [38-40,43]. Accordingly, our study also found that using the KhunLook mobile app preserves these positive effects, including convenience and high acceptance by parents.

### Clinical and Public Health Relevance

This study provides several important findings: using the KhunLook app to assess child’s health can improve parents’ HL by increasing the proportions of parents who have high HL from baseline. This increase is specifically significant for parents’ total HL and health management and child health management domains. Parents reported higher convenience in using the intervention in all domains. These results indicate that the KhunLook app has the potential to be a valuable tool to promote healthy childcare for parents. The intervention can be easily scalable and cost-effective.

### Conclusions

The proportions of parents with high HL category significantly increased for total HL and the child health management and health management domains in the intervention group. Besides, the HL scores improved after the intervention for total HL and 4 domains in both groups. Our results suggest the potential of a smartphone app (KhunLook) to improve HL as well as to promote superior accuracy of parents’ assessment of their child’s head circumference and development, all with a similar effect on weight, height, nutrition and feeding, and immunization as in traditional interventions. Thus, this intervention, KhunLook app, has the potential to be useful and more convenient for promoting a healthy child preventive care during early childhood in families.

## Appendix

Multimedia Appendix 1 CONSORT eHEALTH checklist (V 1.6.1).

Multimedia Appendix 2 Proportions of parents' health literacy by category before and after the intervention by subgroup.

## Abbreviations

HL: health Literacy
MCHH: Maternal and Child Health Handbook
mHealth: mobile health
CONSORT: Consolidated Standards of Reporting Trials
