# Supplementary material for: Effectiveness of a Mobile App (KhunLook) Versus the Maternal and Child Health Handbook on Thai Parents’ Health Literacy, Accuracy of Health Assessments, and Convenience of Use: Randomized Controlled Trial
Source: J Med Internet Res. 2023 May 9;25:e43196. doi: 10.2196/43196 (PMC10206628; doi:10.2196/43196)
Supplement: Multimedia Appendix 2 [file jmir_v25i1e43196_app2.pdf]

Table S1. Proportions of parent's health literacy by category before and after the intervention by subgroup

	Parents of the 1st child						Parents have more than 1 child						Single parents						Parents who are men					
	KhunLook app group n=120			MCHH group n=107			KhunLook app group n=62			MCHH group n=69			KhunLook app group n=22			MCHH group n=22			KhunLook app group n=17			MCHH group n=25		
**Health literacy domain**	Δ High HL*		*P*-value	Δ High HL*		*P*-value	Δ High HL*		*P*-value	Δ High HL*		*P*-value	Δ High HL*		*P*-value	Δ High HL*		*P*-value	Δ High HL*		*P*-value	Δ High HL*		*P*-value
	n	%		n	%		n	%		n	%		n	%		n	%		n	%		n	%	
**Total health literacy**	15	12.5	0.0112	5	4.6	0.3359	0	0.0	1.0000	3	4.3	0.4669	2	9.1	0.3173	3	13.7	0.2568	-1	-5.9	0.5637	-2	-8.0	0.4795
Access to health information and services	7	5.9	0.1779	-2	-1.9	0.7389	5	8.1	0.9167	12	17.4	0.0027	1	4.5	0.6547	4	18.2	0.1573	3	0.0	0.1797	0	0.0	1.000
Understanding of health information and services	-2	-1.7	0.6831	0	0	1.0000	-2	-3.2	0.5637	2	2.9	0.6547	0	0.0	1.0000	-2	-9.1	0.3173	3	0.0	0.0833	1	0.0	0.3173
Health information services and appraisal	0	0	1.0000	4	3.7	0.4142	-3	-4.8	0.4053	4	5.8	0.3938	-2	-9.1	0.3173	0	0.0	1.0000	1	0.0	0.5637	2	0.0	0.4795
Communication and social support	12	10	0.0396	9	8.4	0.1172	1	1.6	0.8273	6	8.7	0.1336	-1	-4.5	0.5637	6	27.2	0.0339	-2	0.0	0.4142	0	0.0	1.000
Health management	26	21.6	0.0001	1	1	0.8658	11	17.7	0.3173	-3	-4.3	0.3711	5	22.7	0.0956	0	0.0	1.0000	3	0.0	0.2568	1	0.0	0.7630
Child health management	11	9.8	0.0555	8	7.5	0.1944	7	11.3	0.1083	2	2.9	0.6374	3	13.6	0.2568	3	13.7	0.3173	-1	0.0	0.6547	0	0.0	1.0000

*Δ High HL= Parents with high health literacy after intervention minus before intervention
Unadjusted P-values are presented, adjusted alpha is considered statistically significant when P-value <0.00625
